# Inducing a Functional-Pharmacological Coupling in the Human Brain to Achieve Improved Drug Effect

**DOI:** 10.3389/fnins.2020.557874

**Published:** 2020-10-09

**Authors:** Roy Sar-El, Haggai Sharon, Nitzan Lubianiker, Talma Hendler, Gal Raz

**Affiliations:** ^1^Sagol Brain Institute, Tel Aviv Sourasky Medical Center, Tel Aviv, Israel; ^2^Sackler Faculty of Medicine, Tel Aviv University, Tel Aviv, Israel; ^3^The School of Psychological Sciences, Tel Aviv University, Tel Aviv, Israel; ^4^Sagol School of Neuroscience, Tel Aviv University, Tel Aviv, Israel; ^5^Steve Tisch School of Film and Television, Tel Aviv University, Tel Aviv, Israel

**Keywords:** drug delivery, brain, functional pharmacology, methylphenidate, ADHD

## Abstract

Neuropharmacotherapy is substantially hindered by poor drug targeting, resulting in low specificity and efficacy. It is known that different behavioral tasks increase functional activity and cerebral blood flow (CBF), two key parameters controlling drug delivery and efficacy. Here, we tested a novel, non-invasive drug targeting approach (termed functional-pharmacological coupling), which couples drug administration with a task that is known to specifically activate the drug’s sites-of-action in the brain. In two studies we administered Methylphenidate (MPH) to neurotypical adults and to subjects with attention deficit hyperactivity disorder (ADHD). In study 1 we employed a within-subject factorial design and found that only following MPH administration, subjects that performed better in the cognitive induction task showed greater improvements in N-back performance. Moreover, only under MPH-Cognitive induction condition, this improvement correlated with concurrent N-Back rDLPFC activation. In Study 2, subjects with ADHD performed better on sustained attention when MPH administration was followed by a cognitive challenge rather than a control task. Again, those who were more attentive to the cognitive challenge scored higher. Our results provide preliminary support for the feasibility of functional-pharmacological coupling concept, hence opening a new horizon for patient-tailored, context-driven drug therapy.

## Introduction

The efficacy of psychopharmacological treatment is often considerably restricted by the fact that drugs reach both pathologically relevant and irrelevant brain areas in a non-selective manner, thus causing desired but also unwanted effects. Thus, a bedside method of enhancing specificity of drug delivery would have important clinical implications. First and foremost, it may allow a reduction in the dosage required for satisfactory therapeutic effects in the target area (i.e., greater efficacy), resulting in reduced abuse and improved side effect profile. In addition, and no less important, it may result in better adherence and reduced economic burden.

In an effort to address the need for better drug targeting, researchers have thus far employed a range of different technological means such as nanocarriers (Khanbabaie and Jahanshahi, 2012), laser stimulation (Nakano et al., 2015), and ultrasound (Hynynen et al., 2001). In this study we present an alternative non-invasive approach, which aims to harness endogenous neurovascular processes to enhance the efficacy of drug delivery in the brain. In specific, we coupled drug administration with the introduction of external psychological stimuli that activate the drug’s target brain regions. We hypothesized that this coupling (termed hereby *Functional Pharmacology*) would result in the enhancement of the expected drug effect.

Our approach relies on the notion that a selective activation of a targeted brain region or circuit could implicate a desired modulation of key pharmacokinetic and pharmacodynamics factors that determine drug delivery effects. Cerebral blood flow (CBF) is a key factor affecting the spread of the drug across the cerebral blood system and ultimately its arrival to target brain sites. Drugs may cross the Blood Brain Barrier (BBB) mostly through the capillaries by processes of diffusion and active transportation. Crucially, blood flow may be the limiting factor for such BBB crossing in the case of flow-dependent substances that are rapidly extracted from the blood (Banks, 2016). Evidence suggests that focal increases in neural activity in the brain in response to various behavioral stimuli\tasks are accompanied by increased local CBF (for a review, see Muoio et al., 2014) with an estimated magnitude of 47–60% (Ito et al., 2001; Chen and Pike, 2010). This effect relies on the neurovascular coupling – a process in which neurons, astrocytes, and vascular cells interact to create local hemodynamic changes. It follows that a behavioral task that selectively activates specific neuronal circuits (i.e., drug’s sites of action) may generate a desired up-regulation in blood flow, and result in increased drug delivery and efficacy.

In the pharmacodynamic domain, Spitzer (2012) demonstrated that neural activity can also modify neurotransmitters expression in the brain, leading to re-specification of receptors. This effect is known to be context-dependent (Inagaki et al., 2012). Hence, both pharmacokinetic and pharmacodynamic factors may be regulated following drug administration in order to improve its delivery and efficacy. In other words, our approach aims to employ psychophysiological means to facilitate optimal pharmacodynamic and pharmacodynamics contexts for efficient drug delivery in the brain. This rationale is analogous to the consideration of the gastrointestinal “context” for better systemic drug absorption; e.g., taking a drug before or after a meal, in the morning or evening.

To demonstrate our new approach, we selected Methylphenidate (MPH), an inhibitor of monoaminergic reuptake, which is commonly used to treat attention deficit hyperactivity disorder (ADHD). Importantly for our aims, MPH absorption in the brain is flow dependent, a characteristic that is measured by the difference between the arterial and venous drug concentration (which is high for MPH). This means that MPH is extracted from the circulation rapidly, accumulated in the BBB and eventually enters the brain (Volkow and Swanson, 2003; Banks, 2016).

While the specific mechanism by which MPH enhances cognitive performance has yet to be fully understood. A recent Positron Emission Tomography (PET) study in humans (Westbrook et al., 2020) suggests that MPH may act on striatal sites to enhance cognitive performance not by boosting the cognitive capability *per se*, but by increasing the motivation to exert cognitive control. This study demonstrates that MPH amplifies the effects of the estimated benefit versus the cost of the required cognitive effort and that this process is mediated by increased attention to benefit cues. At the system level, accumulating evidence points to the importance of MPH influence on the interplay between the mesocortical and the mesolimbic pathways in the brain (for a review, see Cropley et al., 2006). MPH allows the demonstration of differential targeting of the dopaminergic system by functional local activation: dopamine in the mesocortical pathway [from the Ventral Tegmental Area (VTA) to the prefrontal cortex] plays an important role in cognitive and executive functions, including working memory, and underlies MPH’s effect on attention (Floresco and Magyar, 2006). The potentiation of this circuit following MPH administration may be enduring. Thus, a recent functional connectivity study in PET (Birn et al., 2019) indicates that increased levels of extracellular striatal dopamine following MPH administration are associated with increased functional connectivity between the head of the caudate and the prefrontal cortex (including the dorsolateral prefrontal cortex) in subsequent resting state conditions. On the other hand, dopamine in the mesolimbic pathway (VTA’s projections to the ventral striatum) has a crucial role in its effects on the reward system, and is responsible for MPH’s side effects on emotional modulation (Marco et al., 2011).

Drawing on this possible differentiation of dopaminergic effects, we sought to examine whether the coupling of MPH administration with a battery of cognitive tasks that are known to be associated with mesocortical activity, would result in increased drug efficacy on post-coupling cognitive performance that is known to involve prefrontal cortex activation. Importantly, animal and human studies have repeatedly demonstrated that effects of dopaminergic psychostimulants, such as cocaine and amphetamines, may vary dramatically depending on the environmental context in which they are administrated (Ritz et al., 1987; Overton and Clark, 1997; Volkow et al., 2001; Swanson et al., 2002). As for MPH in specific, Volkow et al. (2002) reported that the drug’s effect on extracellular dopamine in the basal ganglia was modulated by the context, so that salient stimuli (visual and olfactory food cues for participants who had fastened for 16–20 h) were associated with higher dopamine increase relative to a neutral task (describing family genealogy). The rationale of this study is in line with the functional-pharmacological coupling hypothesis suggested here, as it was explicitly motivated by the notion that since salient stimuli activate dopamine cells (Schultz, 1998), MPH will interact with the environmental stimulation. However, since dopamine levels were measured during the tasks, this design complicates the interpretation of the results. It is possible that the task itself, rather than the interaction between the task and the MPH, was responsible for the observed effect.

To examine whether the MPH effect on cognitive performance is modulated by the context, we separated the induction task from the probing task and conducted two studies: in Study 1 we pursue evidence for the behavioral effect of MPH functional-pharmacological coupling and its sustained neural markers. In Study 2, we examined the clinical benefit of MPH functional-pharmacological coupling for Attention-deficit/hyperactivity disorder (ADHD) patients.

## Study 1: Neurobehavioral Effects of MPH Functional-Pharmacological Coupling

### Introduction

Based on previous neuroimaging data, we assumed that the activity of mesocortical structures would be enhanced by our cognitive induction protocol (MPH-Cognitive), which includes inhibitory control (Luijten et al., 2014; Constantinidis and Luna, 2019), attention (Knudsen, 2018; Nani et al., 2019), working memory (Emch et al., 2019), and abstract reasoning (Hobeika et al., 2016) tasks. To test the specificity of the effect of MPH-Cognitive task, we added a control task induction condition (MPH-Control) in which MPH administration was coupled with a task known to activate mainly mesolimbic areas, and particularly the ventral tegmental area and ventral striatum (Gonen et al., 2016). To control for overall task effects (beyond the drug effect) we added a placebo drug condition for each task (Placebo-Cognitive, Placebo-Control).

Before and after all four conditions the participant performed a commonly used executive function N-Back task under fMRI scan. Since MPH treatment in ADHD has been consistently associated with increased activation in the prefrontal cortex (Berridge and Devilbiss, 2011), and since the right dorsolateral prefrontal cortex (rDLPFC) was specifically shown to be related to cognitive load in executive functioning (Knoch and Fehr, 2007) such as in the N-Back task (Owen et al., 2005), it was *a priori* defined as a region of interest (ROI). It should be emphasized that the selection of the rDLPFC as out ROI was not motivated by a hypothesis that the functional-pharmacological effect will produce *direct* effect of MPH on dopamine receptors at this site. We expected that the functional-pharmacological coupling would inflict direct and indirect effects across the entire mesocortical pathway. In light of the involvement of the DLPFC in performance of the cognitive task we employed (Berridge and Devilbiss, 2011), and considering evidence that MPH may produce sustained increases in extracellular levels of dopamine (Berridge et al., 2006) and indirectly stimulate D1 receptors in the prefrontal cortex (Gamo et al., 2010), we regard rDLPFC activation as a relevant neural index for functional-pharmacological coupling in this case.

Our specific hypotheses were as follows: 1. Selectively coupling MPH administration with a cognitive task that assumingly activates the mesocortical pathway, would result in greatest improvement in executive function performance, as measured by the N-back task during scanning, compared with the other conditions (i.e., Placebo-Cognitive, MPH-Control, Placebo-Control); 2. The pre-post improvement in N-back performance will be higher among participants that were more engaged in the cognitive task, but only when MPH (rather than placebo) was administered. 3. The improved performance during scanning would correlate with the change in task activity in rDLPFC following drug administration, only in the MPH-Cognitive condition; 4. The increased activity in the rDLPFC following drug administration in the MPH-Cognitive condition would correlate with the performance in the coupled cognitive induction task.

### Materials and Methods

Seventeen healthy participants were recruited via advertisement in social media. Two participants were excluded due to technical problems. Two participants completed only two out of the four sessions due to personal reasons. Thirteen participants therefore completed the study (Male = 10, mean age ± SD: 25.83 ± 5.46; for all statistical analyses *n* = 13). Since participants were students or former students, we assumed normal cognitive function. Participants reported no current use of psychoactive medications or illicit drugs, and had no family history of major neurological or psychiatric disorders. All participants provided written informed consent approved by the Tel Aviv Sourasky Medical Center’s Ethics Committee. Participants were screened by the Adult ADHD Self-Report Scale (ASRS)**;** six questions regarding ADHD symptoms, providing a summary score of 0–24, where 14–24 indicates ADHD (Kessler et al., 2005). All participants were in the 0–14 score range, and the average ASRS score in the group was 13.15 ± 1.21 (*n* = 13).

**FIGURE 1:**
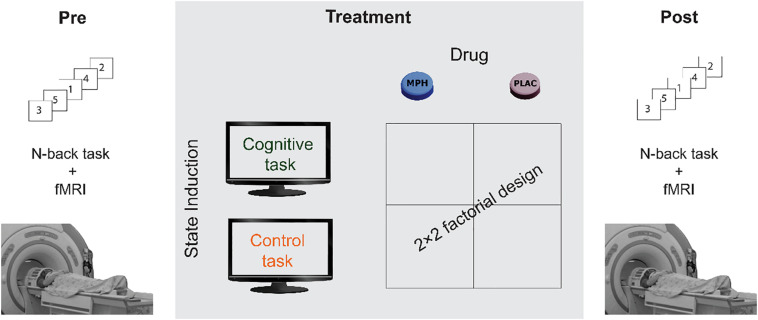
A schematic description of the experimental design. Participants underwent four functional pharmacology counterbalanced sessions in a within-subject 2 × 2 factorial design, resulting in four coupling conditions: Cognitive-MPH, Cognitive-Placebo, Control-MPH, and Control-Placebo. fMRI scanning during the N-Back task was performed before the drug-task coupling procedure and immediately afterward.

The participants underwent four experimental sessions in a double-blind, counterbalanced, within-subjects factorial design, with sessions interspersed by at least a week. In each session, participants received either 30 mg MPH (P.O) or an identical looking starch pill as a placebo. Previous studies that used oral MPH have shown that 20–40 mg corresponds to ∼50% dopamine receptor occupancy (Volkow et al., 2001, 2002; Volkow and Swanson, 2003), a suitable dose to avoid ceiling effect. Drug administration (MPH or Placebo) was followed by a short 15 min break, after which subjects performed either a cognitive or a control task, resulting in four conditions: MPH-Cognitive, MPH-Control, Placebo-Cognitive, and Placebo-Control (Figure 1). Both tasks were applied in a quiet room and lasted approximately 45 min. All sessions were conducted at approximately the same time in the morning. Participants were instructed to refrain from consuming caffeine and alcohol for 24 h prior to each session and this was verified verbally at the beginning of each session.

#### Pharmacological Experimental Parameters

In order to set the correct dose for the unveiling of the functional-pharmacological coupling effect, we relied on the current literature. Previous studies which used oral MPH have shown that 20–40 mgs correspond to ∼50% dopamine receptor occupancy (Volkow et al., 2001, 2002; Volkow and Swanson, 2003), a suitable dose to avoid ceiling effects.

Another important parameter is the time interval between drug administration and the induction task and the concurrent post N-Back task. Even though MPH reaches peak plasma concentration after 1.5–2 h, its concentration in the brain peaks faster, reaching a maximum approximately 1 h after administration, as indicated both by animal and human studies (Volkow et al., 1998) in addition to MPH brain pharmacokinetics, it is also important to take into consideration its transporter occupancy as a function of time. Spencer et al. (2006) show that 1 h following MPH administration, transporter occupancy already reaches its maximum. Additionally, clinical effects following MPH administration reaches its maximum after 1.5–2 h (Swanson and Volkow, 2002). Therefore, we speculated that in order to avoid physiological ceiling effects it is wiser to perform the induction task before maximum concentration and transporter occupancy has reached its maximum; i.e., within the second half of the first hour following drug administration. This has also enabled to conduct the post-test N-Back task when clinical effect is maximal [approximately 1.5 h following administration, when clinical effects are considered highest (Swanson and Volkow, 2002)].

#### Induction Tasks

For the cognitive state induction we used a validated battery of seven computerized cognitive tasks (NeuroTrax Corp., Bellaire, TX, United States), known to probe executive functions, including the following tests: Go-NoGo, Problem solving, Stroop interference, Non-verbal memory, Staged information processing, and Visual spatial processing (Schweiger et al., 2003). Test scores for accuracy and response time (RT) were normalized to a standard scale with a mean of 100 and standard deviation of 15, based on normative data from a large cohort (*n* = 1569) of cognitively healthy individuals provided by the NeuroTrax manual. A global cognitive score, which is the default measure of this battery, was computed as the average of all test scores for a single administration of the cognitive battery. In addition, an “attention index” was computed based on the RT in attention tasks sensitive to ADHD: Go-NoGo, staged information processing and Stroop interference (Schweiger et al., 2003). We chose this index since it probes our cognitive domain of interest by which the ADHD model was selected.

The control condition consisted of two paradigms known to probe reward processing and mesolimbic activation: a 25 min competitive computer game developed in our lab as a paradigm to assess sensitivity to reward and punishment (Gonen et al., 2016), and a 20 min passive listening excerpt of emotional music extracted from the Pachelbel’s Canon, previously used to induce positive emotions (Knight and Rickard, 2001).

Both cognitive and control tasks were applied in a quiet examination room and lasted approximately 45 min. All sessions were conducted at approximately the same time in the morning. Participants were instructed to refrain from consuming caffeine and alcohol for 24 h prior to each session and this was verified verbally at the beginning of each session.

#### N-Back Task

The N-back task performed was composed of three conditions: 0, 2, and 3 back, interspersed with periods of no stimuli. This is a trial of 5.15 min with nine alternating blocks of the three conditions and 11 baseline blocks. Conditions were presented following instructions as a series of one digit numbers. Each block consisted of 10 stimuli. Stimuli presentation rate was 2 s (1 s for a digit interposed with 1 s blank). There were 21 s of no stimuli at the onset and 6 s at the end of the paradigm. Six-second resting epochs were introduced between blocks. During the 0-back conditions, subjects were required to indicate with a gentle finger tap whenever the number 9 appeared on the screen. During the 2-back condition, subjects were required to indicate when the same digit appeared 2 steps before (e.g., 4 6 8 6 2 3) while during the 3-back condition, subjects were required to indicate when the same digit appeared 3 steps previously (e.g., 2 3 5 2 1). In the baseline condition, subjects were instructed to concentrate on the red fixation point in the middle of the screen.

The N-Back task was performed during fMRI scanning before, and 60–90 min following the drug-task coupling procedure on each experimental day. The time interval between drug administration and scanning corresponded to the expected time-to-peak brain concentration of MPH after a single oral administration (Swanson and Volkow, 2002; Balcioglu et al., 2009).

#### Mediation Analysis

To gain further insight regarding the mechanism of functional-pharmacological coupling, we applied a mediation analysis following a standard three variable path model according to the INDIRECT procedure of SPSS (Preacher and Hayes, 2008). The mediation analysis was performed on three variables from the MPH-Cognitive condition: the change in 3-back RT (pre-post) during scanning; the corresponding change in rDLPFC activity (beta values) during the 3-back condition; and the “attention index” obtained from the cognitive induction task that was coupled with MPH. The indirect effect was considered significant if its 95% bootstrap confidence intervals from 10000 iterations did not include zero at *p* = 0.05.

#### Brain Imaging

Prior to the fMRI scan, all participants underwent a preparatory session to verify adequate performance. For the fMRI N-Back task, participants were fitted with a response box and were directed to press the button when appropriate. Participants’ reaction times (RTs) and accuracy rates were collected.

Brain scanning was performed on a GE 3T Signa HDxt MRI scanner with an eight-channel head-coil. Functional images were acquired using a single-shot echo-planar T2^∗^-weighted sequence. The fMRI was acquired during block-design N-back task; a working memory task known to involve the right DLPFC in correspondence to cognitive load (Bleich-Cohen et al., 2014; Thaler et al., 2015). The following scanning parameters were used: TR/TE: 3000/35; flip angle 90; FOV: 20 cm × 20 cm; matrix size: 96 × 96; 39 axial slices with 3 mm thickness and no gap covering the entire brain. Acquisition orientation was of the fourth ventricle plane. In addition, each functional scan was accompanied by a three-dimensional scan using T1-SPGR sequence (1 mm × 1 mm × 1 mm).

#### fMRI Analysis

Preprocessing included correction for head movements (the exclusion criterion was movements exceeding 2 mm or 2 degrees in any of the axes), realignment, normalizing the images to Talairach space and spatial smoothing (FWHM, 6 mm). The first six functional volumes, before signal stabilization, were excluded from analysis.

Functional data were preprocessed and analyzed using BrainVoyager QX version 2.6 (Brain Innovations Maastricht, Netherlands). The rDLPFC ROI was defined based on activity coordinates obtained from an N-back fMRI task previously performed on healthy participants in our lab (Thaler et al., 2015). We converted the statistical activation map into clusters of activation restricted by a threshold of 0.001 (Bonferroni-corrected for multiple comparisons); number of voxels for the rDLPFC ROI was 10177. Whole brain statistical maps were prepared for each participant in each of the eight imaging sessions (Pre/Post for MPH/Placebo-Cognitive, MPH/Placebo-Control) using a general linear model (GLM), in which the N-back conditions were defined as predictors (0-, 2-, and 3-back). In order to conduct the ROI analysis, beta values were extracted from the predefined rDLPFC area, and averaged across all voxels per participant, in each session.

#### Statistical Analyses

As this study investigated a therapeutic effect that has not been tested before, we could not rely on prior results. Therefore, we chose to apply a within-subject design with improved statistical power (compared with the more prevalent between-subjects design), and to follow the common practice in fMRI pharmacological studies regarding sample sizes (group sizes ranging from 12 to 20 subjects; for example, see Domes et al., 2007; Rubia et al., 2011a,b; Smith et al., 2013; Abdallah et al., 2017; our initial sample size = 17).

The relation between brain activity and behavioral performance during high cognitive load was examined for each session by correlating the change in RT (millisecond) with the corresponding change in the rDLPFC activity (beta values) during the 3-Back condition. The relation between the cognitive induction and the functional-pharmacological coupling behavioral outcome was relevant only for MPH/Placebo-Cognitive sessions, and it was tested by correlating the change in 3-back RT (pre-post) during scanning with the performance on the cognitive induction task that was coupled with drug administration.

We directly compared the correlations across the four conditions between pre-post N-back RT change on the one hand and the average rDLPFC ROI beta on the other hand using the method suggested in Raghunathan et al. (1996) for comparison of correlated but non-overlapping correlations. Similarly, we used the same test to compare the MPH-Cognitive and Placebo-Cognitive conditions in terms of the correlations between the pre-post N-back RT difference and the NeuroTrax attention index and global scores. Finally, we compared the correlation between the NeuroTrax measures and the N-back RT change in the MPH-Cognitive condition with the correlation between the NeuroTrax in the MPH-Cognitive condition and the N-back RT change in the MPH-Control condition. This comparison was conducted using a method for comparing correlated correlation coefficients as described in Meng et al. (1992).

### Results

#### Induction Tasks Scores

The validity of the notion of functional-pharmacology was evaluated by analyzing the performance in each task coupled with either MPH or placebo. Cognitive performance in executive functions tasks was measured according to the NeuroTrax attention index, which did not differ between MPH and Placebo [97.216 and 100.441, respectively, *t*(12) = −1.04, *p* = 0.32], suggesting that the cognitive challenge was similarly effective in both drug conditions. In the control task, the motivation index was calculated as the improvement in game score from session 1 to session 4. Game scores were higher in the MPH condition than in the placebo condition [402.167, 282.833, respectively, *t*(12) = 3.0828, *p* = 0.01], suggesting an advantage for MPH in this task.

#### N-Back Behavioral and Neuroimaging Analysis

The effect of functional-pharmacological coupling on the drug’s effect was evaluated by assessing the N-back performance (RT and accuracy) and rDLPFC activity. We first examined differences between conditions in performance prior to all drug-task couplings. There were no differences in the 3-back RT [*F*(3,36) = 0.331, *p* = 0.803] and in the 2-back RT [*F*(3,36) = 0.207, *p* = 0.891] between the four conditions. Then, we validated the cognitive-load effect of the N-Back task across drug conditions and time points in behavioral and brain measures. There was an expected behavioral effect of cognitive-load, both on RT and accuracy measures across drug conditions and time points. The RT for 0- 2- 3-back was 437.35 ± 16.67, 553.79 ± 43.13, and 645.49 ± 61.19 ms, respectively [repeated measures ANOVA, *F*(2,24) = 7.456, *p* < 0.01, 0 vs. 2 back *p* < 0.05, 0 vs. 3 back *p* < 0.01, 2 vs. 3 back *p* < 0.05, Bonferroni-corrected]. The mean accuracy for the 0- 2- 3-back was 96.47 ± 1.5, 86.00 ± 3.21, and 68.57 ± 5.36 correct responses rates (“hits” percent), respectively (repeated measures ANOVA *F*(2,24) = 16.832, *p* < 0.001; 0 vs. 2 back *p* < 0.01, 0 vs. 3 back *p* < 0.001, 2 vs. 3 back *p* < 0.001, Bonferroni-corrected). These behavioral results confirmed that the N-back task indeed manipulated working memory with differential cognitive load effects.

**FIGURE 2:**
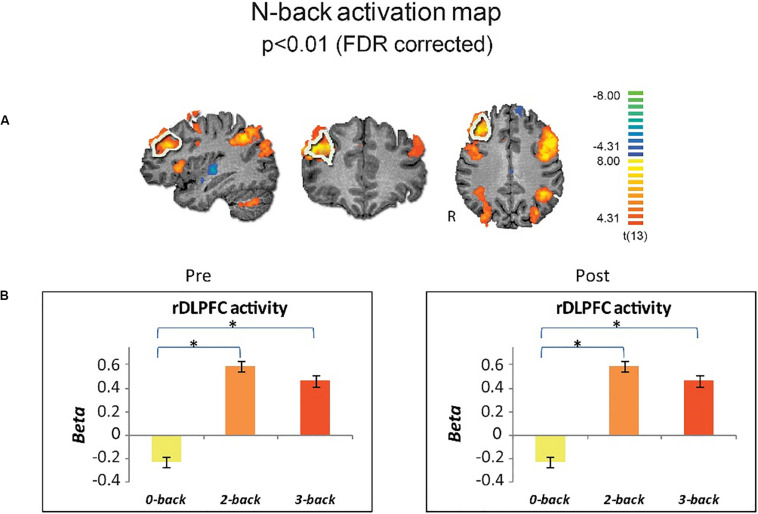
Cognitive load effect on brain activity. **(A)** Group brain activation maps (*n* = 13) for 3-back vs. 0-back are shown in sagittal coronal and axial views (random effects, *p* < 0.01, FDR corrected). The *a priori* selected ROI in the rDLPFC is marked by white boundaries. **(B)** Activity in the rDLPFC shows similar cognitive load-effect for pre- and post drug administration across induction conditions. Error bars stand for Standard Error of the Mean. *p < 0.001.

For a similar cognitive load effect in brain activity, we first obtained whole-brain activation maps, across drug conditions and time points, for the 3-back versus 0-back. As expected, there was greater activity for 3-back than 0-back (*p* < 0.01, FDR-corrected), encompassing typical executive function activity in fronto-parietal areas including the right DLPFC (Figure 2A). ROI analysis for the rDLPFC further showed this effect [Repeated measure ANOVA *F*(2,24) = 74.11, *p* < 0.001], with 0-back showing lower activity than 2- and 3-back, for each time point (*p* < 0.001 for both, Bonferroni-corrected) (Figure 2B).

#### Functional-Pharmacological Coupling – Main Effects and Interactions

To assess the effect of the functional-pharmacological coupling we first looked for changes in the N-back task performance (RT and accuracy) and rDLPFC activity (beta values), using a 2-way repeated-measures ANOVA with drug (MPH/Placebo) and induction task (cognitive/control) as factors during the 3-back load condition. Neither main effect for induction task or drug, nor interaction between factors were found for behavioral [drug main effect: *F*(1,12) = 0.234, *p* = 0.637, induction task main effect: *F*(1,12) = 0.004, *p* = 0.953, drug × task interaction effect: *F*(1,12) = 2.637, *p* = 0.130] or neural measures [drug main effect: *F*(1,12) = 1.5, *p* = 0.244, induction task main effect: *F*(1,12) = 0.018, *p* = 0.895, drug × task interaction effect: *F*(1,12) = 0.6430, *p* = 0.443]. No significant main effect was found on accuracy. The drug main effect was marginal [*F*(1,12) = 3.157, *p* = 0.1], and the induction task main effect was insignificant [*F*(1,12) = 0.005, *p* = 0.947]. The drug by task interaction effect was not significant as well [*F*(1,12) = 0.183, *p* = 0.677].

#### Functional-Pharmacological Coupling – Correlation Analyses

**FIGURE 3:**
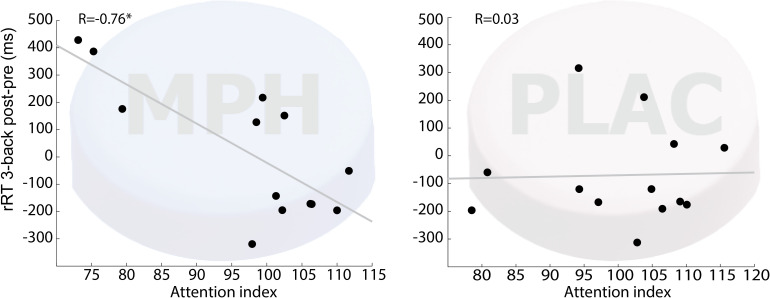
Correlation between attention index during the induction phase and 3-back RT difference (post vs. pre drug-task coupling). Performance on cognitive induction task coupled with MPH, but not with placebo, showed a significant correlation with 3-back RT change during scanning (*R* = –0.76; *p* < 0.01). MPH-Cogni. *p < 0.05 and **p < 0.01.

To inquire the possibility of individual variability in functional-pharmacological coupling effect, we examined whether the behavioral improvement following drug-task coupling corresponds to subjects’ performance during the cognitive induction task, and whether this is evident specifically in the MPH condition. This follows hypothesis 2 that given the presence of MPH in the brain, better performance during cognitive induction would recruit cognitively relevant brain regions and result in greater improvement in the N-Back working memory task. Figure 3 shows that the attention-index derived from the NeuroTrax battery was higher among individuals who showed higher improvement in RT on 3-Back following MPH (*r* = −0.76, *p* < 0.01), but not placebo (*r* = 0.01, *p* = 0.97). A direct comparison of the correlation coefficients indicated a significantly stronger relationship between the variables for the MPH-Cognitive state coupling relative to the Placebo-cognitive coupling (*Z* = −2.4, *p* = 0.016). Similar results were found for NeuroTrax global score as the correlation between the variables was significant under the MPH-Cognitive condition was significant (*r* = −0.69, *p* < 0.01) and insignificant under the Placebo-cognitive condition (*r* = 0.32, *p* = 0.24). The difference between the correlations was significant as well (*Z* = −3.14, *p* = 0.002).

We further examined whether the correlation between the attention index and the N-back RT change in the MPH-Cognitive condition reflects a general tendency of participants with higher attention to exploit training effects possibly facilitated by MPH between. We found that the correlation between the NeuroTrax attention index and the pre-post N-back RT difference in the MPH-Cognitive condition was significantly stronger than the correlation between the attention index in the MPH-Cognitive condition and the N-back RT difference in the MPH-Control condition (*Z* = −2.45, *p* = 0.014). The same was true for the global NeuroTrax scores (*Z* = −1.92, *p* = 0.05). Thus, in line with our hypothesis, this finding suggests that the correlation between the performance in the induction task and the post-pre performance improvement is not based on a general state-unspecific tendency of certain participants (who are also better in attention tasks) to benefit from MPH.

**FIGURE 4:**
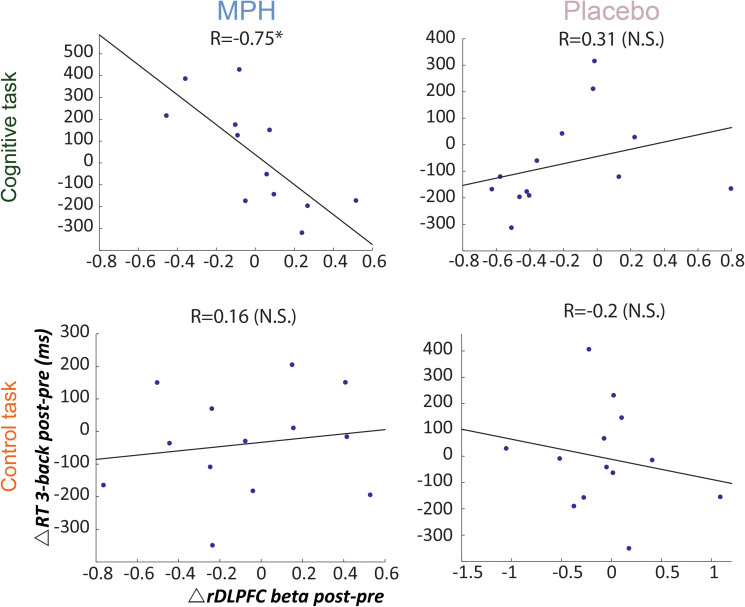
Brain-behavior correlations for the 3-back load, with respect to drug administration and coupling condition. Relationship between change in RT (post vs. pre) and rDLPFC activity (post vs. pre) during the 3-back, following each coupling intervention (MPH-Cognitive, Placebo-Cognitive, MPH-Control, and Placebo-Control). Only the MPH during cognitive task revealed a significant correlation (*r* = –0.75, *P* < 0.01). *p < 0.05 and **p < 0.01.

Next we investigated individual variability for drug effect by correlating between RT and rDLPFC activity during the 3-back load condition, per functional-pharmacology coupling condition. Figure 4 shows that only during the MPH-Cognitive condition there was a significant correlation between change in rDLPFC activity (Post > Pre drug) and RT (Post < Pre drug), indicating that greater increase in rDLPFC activity corresponded with greater reduction in RT following MPH (*R* = −0.75, *p* = 0.003; left upper panel). Direct comparisons indicated that the correlation in the MPH-Cognitive condition was significantly larger than in the Placebo-Cognitive (*Z* = −2.89, *p* < 0.05, Bonferroni-corrected) and the MPH-Control (*Z* = −2.68, *p* < 0.05, Bonferroni-corrected) conditions.

In addition to the main analysis performed with the most difficult N-back condition, the 3-back, we calculated the 2-back post-pre difference for the experimental sessions. We then correlated this difference with the NeuroTrax cognitive induction score (in the same manner reported for the 3-back condition in the main text). Similar to the 3-Back results, the attention-index derived from the NeuroTrax battery was higher among individuals who showed greater improvements in RT on 2-Back following MPH, although this correlation was not significant (*r* = −0.34, *p* = 0.26), indicating that stronger functional-pharmacological coupling resulted in improved performance. Regarding the rDLPFC brain activity (post-pre difference, following the MPH + cognitive induction condition), when averaging the 2-back and 3-back beta values as a combined measure of the rDLPFC improvement in recruitment, we found a significant correlation (*r* = −0.63, *p* < 0.05) to the corresponding averaged 2-Back and 3-Back RT, which was not found for any other condition in the experiment – as is portrayed by using only the 3-Back condition.

We repeated the 2-back and 3-back analyses with accuracy instead of RT data. While the correlation between the rDLPFC beta and the accuracy pre-post change was positive in the MPH-Cog condition, it did not reach the significance level for 2-back (*r* = 0.41, *p* = 0.17) and 3-back (*r* = 0.42, *p* = 0.15). Similarly, we observed negative correlations between the general NeuroTrax attention index (*r* = −0.17, *p* = 0.59) and the attention scores (*r* = −0.24, *p* = 0.41), but these correlations were not significant.

#### Mediation Analysis

**FIGURE 5:**
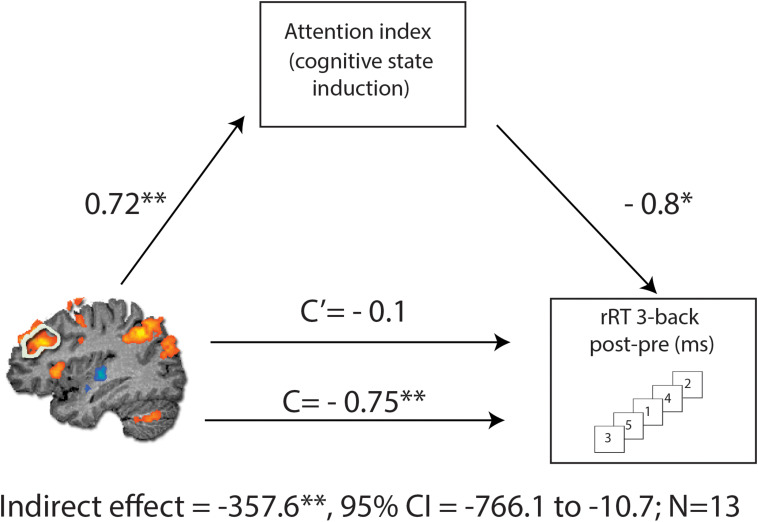
Mediation analysis, describing possible causal mechanisms by examining variables that partially or fully account for the relationship between two variables. C represents the direct effect of rDLPFC beta difference on change in RT for the 3-back condition, while C’ represents the indirect effect, when including the NeuroTrax attention index in the model. Including the attention index in the model results in a non-significant effect (C’), consistent with the mediation hypothesis. The indirect effect was tested using a bootstrap approach with 10000 samples. The model is considered significant since its 95% bootstrap CI from 10000 iterations did not include zero at *p* = 0.05. *p < 0.05 and **p < 0.01.

To assess causal relations in the observed association between change in the rDLPFC activity and RT in 3-back condition following MPH, we performed a mediation analysis (Preacher and Hayes, 2008) with the attention index of the cognitive induction as a mediator. As expected, we found a significant indirect path from rDLPFC activity and RT improvement through the performance during the cognitive induction coupled with MPH administration [indirect effect = −357.6, SE = 198.67, CI (95%) = −766.1 to −10.7] (Figure 5). This effect was not found for the placebo condition [indirect effect = −24.26, SE = 84.39, CI (95%) = −450.78 to 73.51], suggesting that attention scores during cognitive induction coupled with MPH mediated the association between rDLPFC beta difference and 3-back RT difference.

### Discussion

Study 1 introduced a novel concept for improved drug targeting in neuropsychiatry, namely the coupling of drug administration and behavioral induction of a specific, functionally relevant, localized neural activity for the optimization of pharmacotherapy efficacy. To examine the potential of this concept in the case of MPH, we coupled the administration of the drug with a demanding cognitive task and tested drug targeting effects with executive function task performance and its related rDLPFC fMRI BOLD activity.

Taken together, our findings suggest that the coupling of MPH administration specifically with a cognitive induction task may improve performance (i.e., drug effect) in a subsequent working memory challenge. However, since at odds with our first hypothesis the average improvement in the MPH-Cognitive condition was not significantly higher than in other conditions, it seems that the beneficial effect does not result from a passive and uniform process inherent to performing the actual task. The correlation comparisons suggest that it may rather depend on the individual performance during the cognitive induction condition (as indicated by the attention index). Our findings also indicate that under drug-functional task coupling a lasting facilitation of rDLPFC recruitment is associated with a corresponding behavioral improvement in the context of high cognitive load. This observed mediation effect points to a possible connection between functional-pharmacological coupling and a sustained prefrontal effect.

While the correlation findings reported here provide initial evidence that relevant context manipulation may enhance drug therapeutic effects through beneficial relevant neural targeting the null findings and limitations should be acknowledged. First, the current study did not directly measure DA activity in the brain, future studies may shed light on the underlying neural mechanisms involved in the functional-pharmacological coupling concept by using PET with specific drug related ligands. Another limitation is that the controlled manipulation, which involves a competitive computer game, possibly also activates to some degree the mesocortical regions since it requires sustained attention and cognitive control of motor performance. Moreover, as suggested by Westbrook et al. (2020), MPH acts on striatal dopamine receptor and previous findings suggest that our control task activates the striatum (Gonen et al., 2016) so it might increase the likelihood of Type 2 (false negative) errors and weaken the main effect of the induction task by drug interaction. However, the activation of the striatum in the test and control conditions diverged in their neural and psychological context. The MPH- Cognitive condition involved primarily working memory and high cognitive loads of attention. These are expected to implicate striatal connections with prefrontal regions along mesocortical dopaminergic pathways. This would not be the case with the task we used in our control condition, which is known to activate modulate mesolimbic [VTA and ventral striatal rather than the caudate nucleus in Westbrook et al.’s (2020) work] regions in a motivational context. At any rate, in study 2, we selected a control condition that does not require interaction and focused attention.

It is important to state that although in both 2 and 3-Back conditions a cognitive load effect was apparent (both behaviorally and neurally, as indicated by behavioral results and rDLPFC activity), weaker correlations with rDLPFC activity and with the induction task scores were found for the 2-Back condition. It is possible that these differences in magnitude and significance, indicate that the functional-pharmacological coupling might be most effective when the cognitive load is relatively high, and less so for moderate cognitive load. However, as our design did not test this systematically, this remains a speculation to be investigated in future studies. Another possible interpretation is that the functional-pharmacological coupling effect was not expressed solely by rDLPFC recruitment, but rather by a more widespread neural pattern including various brain regions – which might explain why even though the 2-Back and 3-Back conditions produced similar responses in rDLPFC, 2-Back does not correlate as well to the rDLPFC or to the cognitive induction scores; in other words, the rDLPFC might not tell “the whole story.” Future research may benefit from focusing on these locality issues and broaden the scope to additional regions.

While we found evidence for correlations of our neuroimaging and behavioral indices with RT, no significant findings were observed when examining the accuracy measures. At this point, it is worth noting that RT and accuracy may not represent the same aspects of cognition. Previous evidence points to the possibility that the accuracy and speed of reaction to attentional cues reflect distinct cognitive and neural processes, as these measures correlated with different indices or conditions (e.g., Kwon et al., 2002; Chan et al., 2015). A systematic examination of this issue (van Ede et al., 2012) suggests that the parameter of reorienting may affect reaction time, but not accuracy. Consequently, it is possible that the specific technique of functional pharmacological coupling employed in our study, specifically influenced RT-related but not accuracy-related aspects of the task. Moreover, even though we did not observe ceiling effects for the accuracy measures, they may still not be sensitive enough due to low variance relative to RT as the crucial property is not the averages of the different levels *per se*, but rather their variance. The error rates in the different N-back conditions was 3.5%, 14%, and 31.4% in the 0, 2, and 3-back conditions, respectively. These measures had lower variance than for the RT measures: when standardizing the variance using Coefficient of Variation (CV; standard deviation divided by the mean), an index that enables to compare between these two measures, the CV for the 0-back, 2-back, and 3-back in the accuracy measure were 0.06, 0.13, and 0.28, respectively, while for the RT measure it was 0.24, 0.37, and 0.41. Since correlations are dependent upon the variance of the measures, the RT was probably more sensitive for such analysis.

Interestingly, game scores (performance in the control induction condition) were higher in the MPH condition than in the placebo administration condition. As MPH acts upon DA mesolimbic regions (Andersen et al., 2008; Mizuno et al., 2013) (which are known to be associated with reward-related processes engaged in this task), it is possible that MPH strengthened control induction task performance relative to placebo administration. This may point to the fact that, in healthy individuals, MPH mesolimbic effects are more expressed than MPH mesocortical effects. However, this was not systematically tested in this study. Moreover, this result did not lead to observable differences between MPH-control and Placebo-control manipulations. Therefore, interpretations should be cautious.

Another limitation of Study 1 is that this study was conducted on healthy participants. Hence, regarding MPH effects on attention and cognition, the lack of behavioral differences between conditions might be due to ceiling effects resulting from normal performance of the participants in the cognitive task which served as our dependent measure. Therefore, a clinical study with ADHD patients is expected to provide additional information and support for the proposed approach.

## Study 2: Clinical Validity of MPH Functional-Pharmacological Coupling: A Pharmacological-Behavioral Study With ADHD-Diagnosed Participants

The aim of this study was to examine the functional-pharmacological coupling effect demonstrated in study 1 in adult-ADHD in terms of improvement in ADHD-specific measures. The specific coupling, we hypothesized, will better target the drug to the mesocortical pathway, thus specifying its effects resulting in better performance in ADHD-specific cognitive tests, and indicating the clinical applicability of the approach.

It should be noted that Study 2 does not aim to replicate Study 1, but rather to allow an assessment of the clinical value of functional-pharmacological coupling. It addresses the following question: given an MPH treatment, would individuals with ADHD benefit from a concurrent cognitive challenge?

### Introduction

Attention deficit hyperactivity disorder is characterized by inattention and/or hyperactivity and impulsivity, as well as executive dysfunction, associated with lifelong functional impairment. Many children diagnosed with ADHD continue manifesting these symptoms to some extent throughout adulthood, with studies reporting 40–60% of ADHD persisting to adulthood (Bonvicini et al., 2016). The prevalence of childhood ADHD is estimated at approximately 5%, and in adults it is estimated at 2.5–5%. Adult ADHD is characterized by reduced levels of hyperactivity and impulsivity, with a shift toward the inattentive feature (Volkow and Swanson, 2013). The hyperactive feature might present itself through restlessness, while the inattentive feature as difficulties in academic and professional tasks. These manifestations are a huge burden on society, both at the individual level and on the social and economic levels. The economic burden of ADHD has been estimated at $143-266 billion dollars annually in the United States, and ADHD-diagnosed adults have been shown to have a lower socioeconomical status and a higher rate of unemployment (Cortese et al., 2018). This is further emphasized by the higher rate of substance use and addiction in ADHD, as well as various psychiatric comorbidities such as depression and anxiety (De Graaf et al., 2008; Profile et al., 2009; Volkow and Swanson, 2013). In the academic domain, students with ADHD are at higher risk for poor academic performance and are less likely to complete their academic course. In addition, they are also prone to have social difficulties and more likely to engage in substance abuse. This is despite protective factors, such as high cognitive ability in ADHD-diagnosed students (for a review, see McCabe et al., 2005).

The pharmacological treatment of ADHD includes psychostimulants such as MPH and amphetamines, as well as non-stimulants such as atomoxetine. While the number of prescriptions for ADHD medications increases each year (Chai et al., 2012), its efficacy is debatable.

Moreover, while MPH is considered a relatively safe drug, it does have several adverse effects. In individuals (both children and adults) maintained on therapeutic doses of MPH, adverse effects such as sleep difficulties, decreased appetite, blunted affect, nervousness, and obsessive thinking have been reported (Leonard et al., 2004). More severe MPH adverse effects are related to its sympathomimetic activity, causing increased heart rate and blood pressure. This is especially important in adult-ADHD, in which comorbidities with cardiac pathologies such as arrhythmias and hypertension are more likely. Although non-cardiovascular adverse effects are usually mild to moderate, and cardiovascular adverse effects are relatively rare (Leonard et al., 2004), the mitigation of these effects by the improvement of MPH mesocortical targeting is of clinical significance.

Study 2 estimates the clinical plausibility of functional-pharmacological coupling in ADHD. Employing a within-subject design, we coupled MPH administration with either a cognitive induction task or a control, non-cognitive task. Participants were all ADHD-diagnosed, taking MPH occasionally, mainly for academic, and professional performance. In this study, the functional-pharmacological coupling effect, as is appropriate for a clinical ADHD sample, was measured by ADHD-specific measures, evaluating two main cognitive domains related to ADHD, namely *response inhibition*, which requires control over responses considering a change in context (Verbruggen and Logan, 2008), and *sustained attention*; i.e., the readiness to detect rare and unpredictable stimuli over prolonged periods of time (Sarter et al., 2001).

We specifically proposed the following hypotheses:

1. Selectively coupling MPH administration with a cognitive induction (MPH-CogniCognitive) will result in better performance in the ADHD-related cognitive domain of sustained attention, as measured by a continuous performance test (CPT), compared with the control condition (MPH-control).
2. Selectively coupling MPH administration with a cognitive induction (MPH-CogniCognitive) will result in better performance in the ADHD-related cognitive domain of response inhibition, as measured by a stop-signal task (SST), compared with the control condition (MPH-control).
3. Performance in the ADHD-related tasks following drug administration in the MPH-CogniCognitive condition, but not in the MPH-control condition, will be correlated with performance during coupling with the cognitive induction task.

### Materials and Methods

#### Participants

Twenty participants were recruited for the study via advertising in social networks (F:13, M:7, mean age 25.68, Std. 2.54). All participants were right-handed, had a valid documentation of ADHD diagnosis by a physician, reported no current use of psychoactive medications or illicit drugs, and had no family history of major neurological or psychiatric disorders. All participants were current or former university students. All participants provided written informed consent approved by the Tel Aviv Sourasky Medical Center’s Ethics Committee to take part in the study and were paid for their participation at the end of the study. Participants filled out the Wender-Utah rating scale for ADHD (Wender et al., 1993), a scale used to assess adults for ADHD, with a subset of 25 questions associated with this diagnosis. Previous reported use of MPH among the participants was between 10 and 20 mg; all participants reported using MPH for academic or work-related purposes and did not take MPH on days free of work or academic courses.

#### Procedure

**FIGURE 6:**
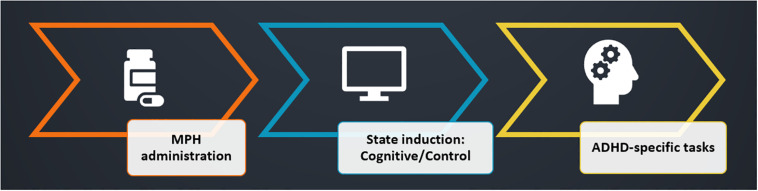
Experimental design. Participants were administered with MPH, coupled with a cognitive/control state induction (session order was randomized). ADHD-specific tests were performed as a measure of the functional-pharmacological coupling effect. Both induction tasks started 15 min following drug administration, and duration was 1 h, followed by a short break. Measurement of the dependent variable (ADHD-specific attention tests) was performed 1.5 h post-MPH administration, at a time when peak MPH levels are expected in the brain.

The study employs a within-subject design, with two experimental sessions in random order interspersed by at least a week (Figure 6). At the beginning of each session, participants received immediate release (IR) MPH (0.25 mg/kg, rounded down to 10/15/20 mg), encapsulated. This dosage was selected in light of the ED50 [the dosage required for a 50% transporter occupancy (Volkow et al., 1998)] for MPH, which was estimated to be 0.25 mg/kg (Volkow et al., 1998). The dosage was rounded down in order to avoid possible ceiling effects caused by high transporter occupancy, which might prevent detecting our experimental effect (Table 1).

**TABLE 1:** MPH dosing frequencies in the study.

**Dose**	***n***
10 mg	7
15 mg	10
20 mg	3
$\bar{X},S$	14.05 (3.39)

*Administered dose was determined as 0.25 mg/kg (Volkow et al., 1998).*

In the experimental condition (cognitive state induction), participants performed the NeuroTrax^®^ cognitive tests battery described in study 1, whereas in the control condition participants watched a relaxing nature movie on the computer screen (scenery only, without human or animal figures). Both tasks started 15 min following drug administration, and duration was 1 h, followed by a short break. Therefore, the measurement of the dependent variable (ADHD-specific attention tests) was performed 1.5 h post-MPH administration, at a time when peak MPH levels are expected in the brain (Volkow et al., 1998; Spencer et al., 2006). Each of the ADHD-specific tests measured a major domain impaired in ADHD. The first test was a CPT, measuring sustained attention (SI). The second test was the Stop-Signal Test (SST), measuring response inhibition (RI). Session order (MPH-Cognitive, MPH-Control) and within-session test order (CPT, SST) were randomized. A total of 10 participants completed the cognitive condition session first, while nine participants completed the control condition first (one participant completed only one session).

#### Wender-Utah ADHD Rating Scale

**FIGURE 7:**
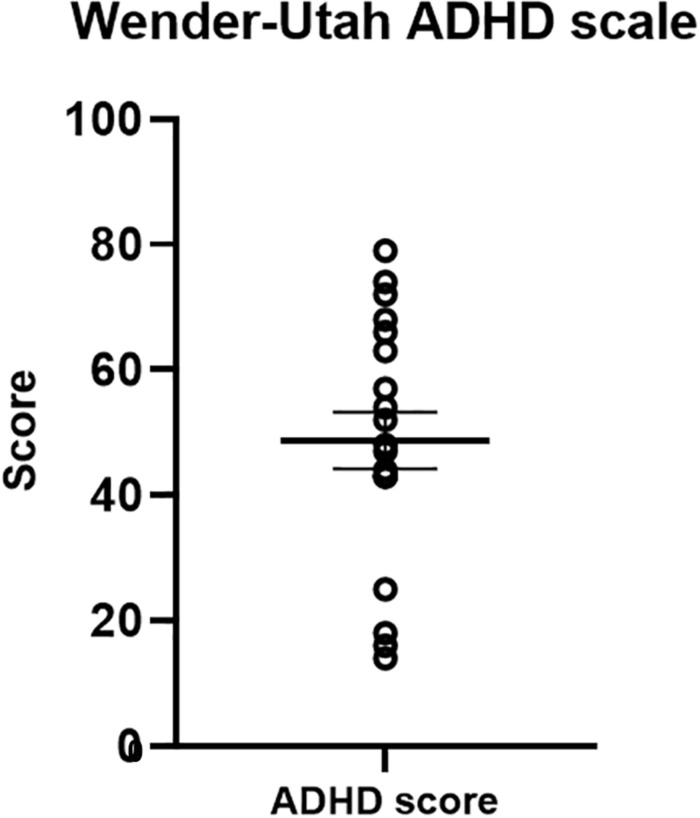
Wender-Utah ADHD scale scores. The mean in the current sample was 50. 76 – higher than a cut-off score of 46, previously found to identify 86% of ADHD-diagnosed adults (Wender et al., 1993).

The Wender-Utah scale served as a validation measure for our ADHD-diagnosed sample (Figure 7). Previous studies have indicated that a cut-off score of 46 in the Wender-Utah rating scale correctly identified 86% of patients with ADHD, and 99% of the normal participants (Wender et al., 1993). Of importance for our clinical sample, the average score of the participants was 50.76 (±19.11) higher than the cut-off value [although not reaching statistical significance in a one-sample *t*-test, *t*(19) = 1.03, *p* = 0.32].

#### Cognitive Induction Tasks

For cognitive state induction, we employed the same validated battery of seven computerized cognitive tasks (NeuroTrax Corp., Bellaire, TX, United States), described in study 1 (Schweiger et al., 2003). Calculation and normalization of test measures, as well as the use of global and domain-specific scores, were performed in the same manner as in study 1.

#### Dependent Measures: Sustained Attention and Response Inhibition Tasks

1. Continuous Performance Test: The CPT is a common measure of attention, especially in the setting of ADHD diagnosis. CPT performance is abnormal in both children and adults with ADHD relative to normal controls, and has been correlated with various ADHD measures such as self-report questionnaires and teacher ratings (Scotia, 1990; Epstein et al., 2010). The task is based on the ability to focus and remain vigilant over time. In the current version of the CPT (Shalev et al., 2011), 320 pictures of geometrical shapes in various colors were presented on the computer screen. Each stimulus was 1.4–1.8 cm in height and 1.8–1.9 in width. Stimuli included 16 different shape-color combinations; each stimulus was presented for 100 ms. The inter-stimulus interval (ISI) was 1,000, 1,500, 2,000 or 2,500 ms (both stimuli and ISI were sampled randomly, each ISI appearing in 25% of the trials). The total duration of the task was 10 min. The participant was instructed to press the space bar with the pointing finger of the dominant hand, as quickly as possible, upon detecting a red square only, and to avoid responding to any other stimulus. The target was presented in 30% of trials (35% of the stimuli are not a square and not red; 17.5% are a non-red square; 17.5% are a red, non-square shape). The CPT has four main outcome measures: mean response time to target stimuli (recorded in milliseconds), standard deviation of the response time to target stimuli, commission error (responding to non-target stimuli) percent, and omission error (not responding to target stimuli) percent. Normal population is characterized by a low error rate of 1–2% (commissions and omissions) and by consistent performance as measured by response time standard deviation, whereas ADHD-participants are expected to show higher inconsistency (Shalev et al., 2011).
2. Stop Signal Task (SST): In this task (Berger et al., 2013), measuring response inhibition, the participant responds to a go stimulus with a discrimination task (one response to stimuli A, another response to stimuli B). In a small proportion of cases (usually 25%), a stop signal appears. When the delay from the go stimulus to the stop signal is long, response inhibition is harder to perform; this is interpreted as a “race” between the “go” and the “stop” processes. Stopping a response requires quick control that prevents the motor response. Dynamic tracking of the participant’s performance is used for setting the delay duration time (Berger et al., 2013). The calculation of the dependent measure in this sort of task is Stop Signal Response Time (SSRT) = mean reaction time in the “go” trials-median dynamic delay time. SSRT represents the latency of the “stop” process and was proven to be a measure of cognitive control processes that are involved in stopping response inhibition. The SSRT was shown to be elevated in children and older adults compared with young adults (Verbruggen and Logan, 2008). In the current study, a visual SST was presented using E-Prime software (PST Inc., E-Prime 2.0) (Hadar et al., 2017). The task included two cue stimuli – “x” and “o.” Participants were instructed to press a corresponding button to each stimulus using the index finger of the dominant hand. In 25% of the trials, the stimuli background color changed from black to white; in such trials, participants were instructed to withhold their response. The Stop Signal Delay (SSD) was performance-related; the duration of the delay from the withhold cue changed dynamically depending on performance.

#### Statistical Analysis

Statistical data analysis was performed using IBM SPSS Statistics for Windows, Version 20.0 (Armonk, NY, United States: IBM Corp.) and GraphPad Prism 7.00 for Windows (GraphPad software, La Jolla, CA, United States).

### Results

#### Cognitive Induction Condition Scores

**FIGURE 8:**
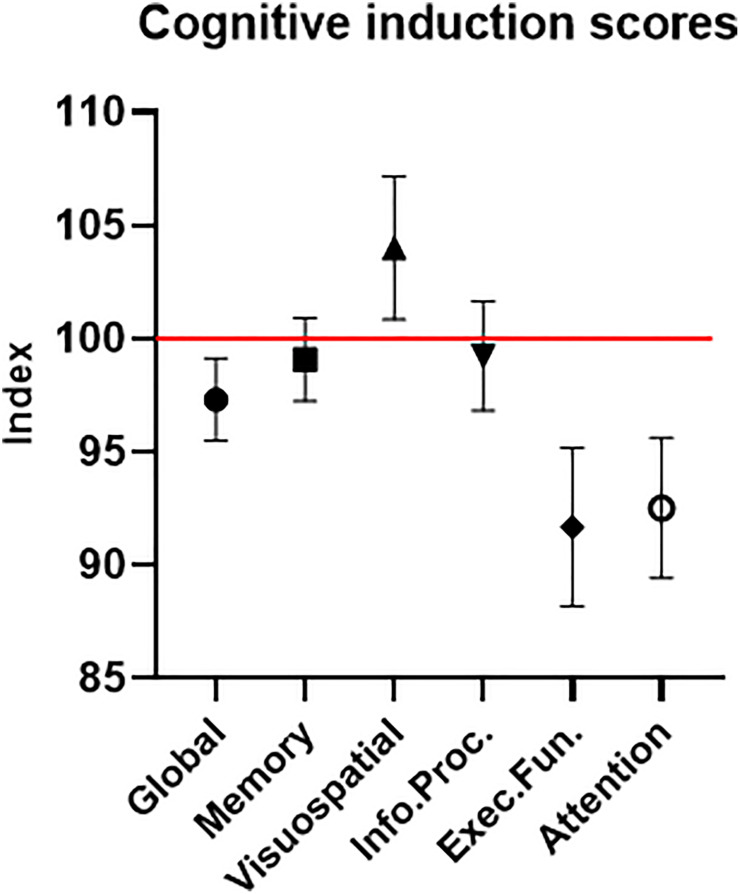
Cognitive induction scores. The global score is comprised of the different cognitive domains’ sub-scores. The red line represents the healthy population’s normalized mean (100). Error bars stand for Standard Error of the Mean. As expected in an ADHD-diagnosed sample, the global cognitive score, as well as most sub-scores, were below the healthy population’s mean.

The participants’ average cognitive global score was 97.31 (±8.16) – lower than the normalized healthy population score of 100 (Figure 8). Of importance for this study and further validating our sample of ADHD-diagnosed adults, this was true for the attention domain average score as well, which was 92.52 (±13.82). The other cognitive domains’ average scores were 99.07 (±8.19) for memory, 104.02 (±14.19) for visuospatial, 99.24 (±10.78) for information processing and 91.67 (±15.69) for executive function. These results help validate our clinical sample of participants regarding two major points. First, the attention scores reflect lower-than-normal attention performance, as expected with ADHD-diagnosed participants. Second, the attention scores provide clear evidence that a ceiling effect, which was possible in a sample with high attention scores, was avoided.

#### Continuous Performance Task

The first step of CPT analysis involved examination of Pearson correlations between variables, as a measure of collinearity, in the different conditions (Table 2). As expected, response time was correlated with standard deviations in both conditions, as were commissions and omissions. Omissions were correlated with standard deviations only in the MPH-CogniCognitive induction condition.

**TABLE 2:** Pearson correlations between CPT variables.

***Cognitive***	**Commissions**	**Omissions**	**RT**	**STD**
Commissions	–	0.59**	0.04	0.40
Omissions	0.59**	–	0.32	0.56*
RT	0.04	0.32	–	0.52*
STD	0.40	0.56*	0.52*	–

***Control***	**Commissions**	**Omissions**	**RT**	**STD**

Commissions	–	0.77**	0.08	0.32
Omissions	0.77**	–	0.08	0.34
RT	0.08	0.08	–	0.75**
STD	0.32	0.34	0.75**	–

* *p* < 0.05, ** *p* < 0.01.

##### Order effects

There was no order effect in CPT measures between the two order conditions: response time [*t*(17) = 1.02, *p* = 0.33)], standard deviation [*t*(17) = 1.89, *p* = 0.08], omission error rate [*t*(17) = −0.82, *p* = 0.42] or commission error rate [*t*(17) = −1.6, *p* = 0.14].

##### Between-conditions differences (Table 3)

**FIGURE 9:**
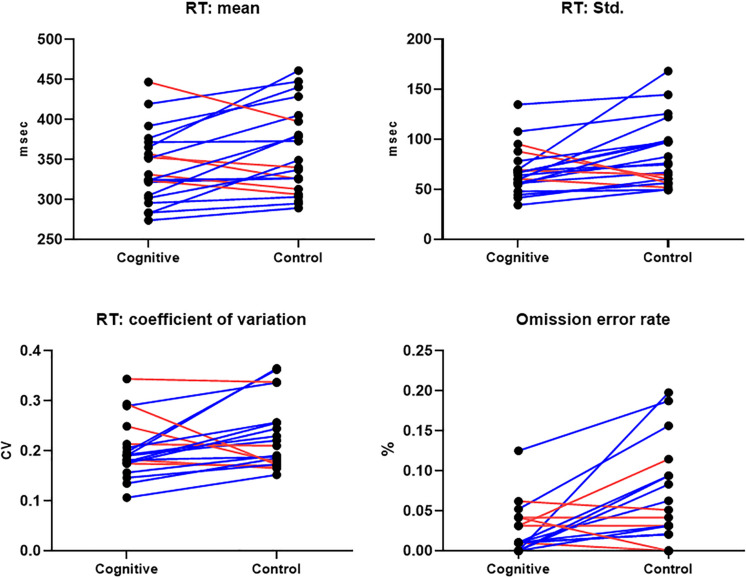
CPT Results, MPH-Cognitive induction condition vs. MPH-Control induction condition. **Upper panel:** Response time mean and standard deviation, per -participant, per -condition. **Lower panel:** Coefficient of variation (standardized measure of dispersion) and omission error rate, per -participant, per -condition. In all presented measures, participants performed better on average in the MPH-Cognitive induction condition compared with the MPH-control induction condition. Blue lines represent participants who performed better in the MPH-Cognitive condition, while red lines represent participants who performed better in the MPH-control condition.

CPT performance measures are presented in Table 3 and Figure 9. The average response time in the cognitive induction conditions was 338.22 ms, and the standard deviation was 68.28. The average response time in the control condition was 357.14 ms, and the standard deviation was 82.81. We performed a paired-samples *t*-test of the difference between conditions in response time (including effect size calculation), which was significant [*t*(19) = −2.15, *p* = 0.045, Cohen’s d = −0.49]. We performed the same test on the standard deviation measure, which was significant as well [*t*(19) = −2.31, *p* = 0.033, Cohen’s d = −0.53]. The average omission rate in the cognitive induction condition was 2.1%, (SE = 0.03), while the average omission rate in the control induction condition was 5.6% (SE = 0.06). This difference was found to be significant in a paired samples *t*-test [*t*(19) = −2.81, *p* = 0.012, Cohen’s d = −0.64]. Finally, the average commission rate in the cognitive induction condition was 1.2% (SE = 0.01), while in the control condition the average commission rate was 1.6% (SE = 0.02). This difference was not significant [*t*(19) = −1.08, *p* = 0.29, Cohen’s d = −0.25]. We also calculated the coefficient of variation (CV), a standardized measure of dispersion of a distribution, defined as the standard deviation divided by the mean. The CV in the cognitive condition was lower than the CV in the control condition (0.19 and 0.23, respectively), and this difference was found to be significant [*t*(19) = −2.14, *p* = 0.045, Cohen’s d = −0.48].

**TABLE 3:** Descriptive statistics for the CPT measures, overall and divided by conditions: MPH-CogniCognitive induction, MPH-control induction.

**Response time (millisecond)**			Standard deviation (millisecond)*			Omission error rate*			**Commission error rate**		
	349.44			76.07			4.2%			1.4%	

**Cognitive**		**Control**	**Cognitive**		**Control**	**Cognitive**		**Control**	**Cognitive**		**Control**

338.22		354.17	68.28		82.81	2.1%		5.5%	1.2%		1.6%

* *p* < 0.05.

#### Stop-Signal Task

Due to a technical problem with task software, SST data was available for 15/20 in the cognitive condition and 14/20 in the control condition. Full data (for both sessions, enabling calculation of between-conditions difference) were available for 13/20 participants. The SST dependent measure was the SSRT, calculated per subject, per session.

**FIGURE 10:**
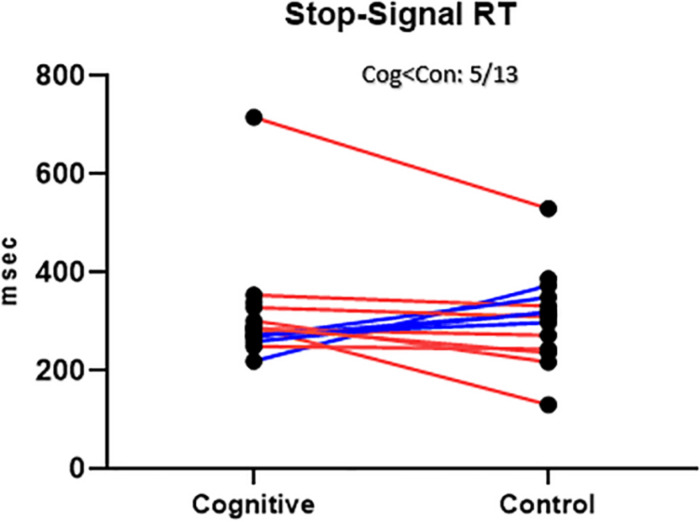
SST Results, MPH-Cognitive induction condition vs. MPH-Control induction condition. Stop-Signal Response Time (SSRT), per -participant, per -condition. There was no significant average difference between conditions in the SSRT. Blue lines represent participants who performed better in the MPH-Cognitive condition, while red lines represent participants who performed better in the MPH-control condition.

There was no significant order effect on the SSRT difference between the two conditions (cognitive, control) [*t*(11) = 1.47, *p* = 0.17; Figure 10].

##### Between-conditions differences

The average SSRT in the cognitive condition was 314.59 (SD = 116.16, *n* = 15). The average SSRT in the control condition was 308.01 (SD = 93.15, *n* = 14). For the 13 participants with full data, the average SSRT was 315.14 (SD = 124.93) in the cognitive condition and 301.92 (SD = 94.00) in the control condition. The difference between conditions was not statistically significant [*t*(12) = 0.51, *p* = 0.62]. This result is at odds with the hypothesis that cognitive induction will result in better response inhibition, the cognitive domain reflected in the SST.

#### Correlations Between Cognitive Induction Scores and Outcome Measures – CPT and SST

For evaluating the correlation between cognitive induction measures (as measured by the NeuroTrax scores) and cognitive performance in the ADHD-related outcome measures – CPT and SST- we calculated the correlations between all measures.

##### CPT

**FIGURE 11:**
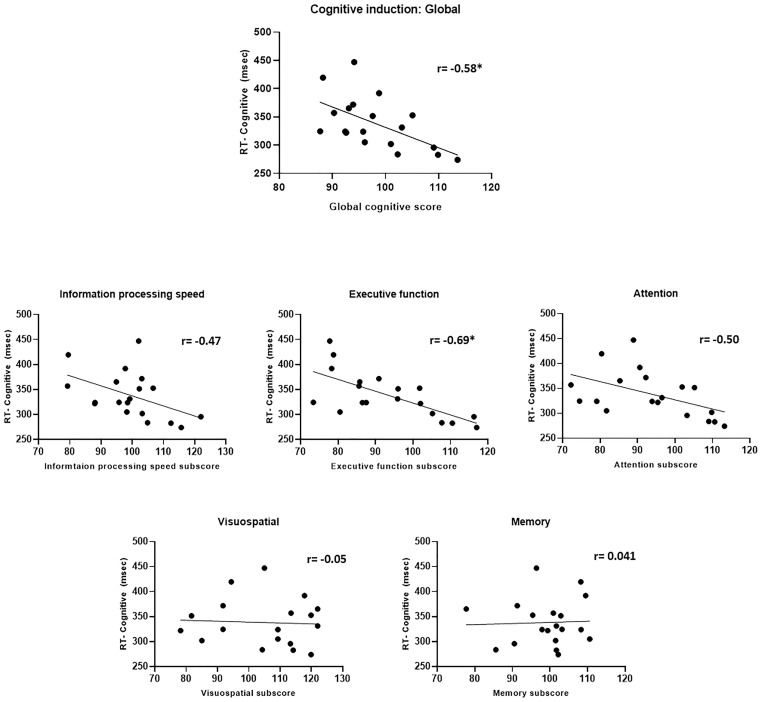
Cognitive induction scores and CPT performance. **Upper panel:** Global cognitive score and CPT response time. **Middle and lower panel:** Cognitive induction battery sub-scores and CPT response time. Better cognitive induction performance in ADHD-related domains (information processing, executive function, and attention) was related to better CPT performance, while performance in less ADHD-related domains (visuospatial and memory) was not related to CPT performance (*p < 0.05, Bonferroni-corrected).

The relationship between performance in the cognitive induction task (the NeuroTrax scores) and CPT scores obtained post-induction showed an intriguing pattern (Figure 11). A better overall global cognitive score correlated with faster CPT response time (*r* = −0.58, *p* = 0.01). When examining the sub-scores in the ADHD-relevant domains of attention, information processing and executive function – better scores were related to faster CPT response time (*r*_attention_ = −0.50, *p* = 0.03, *r*_Info.proc._ = −0.47, *p* = 0.04, *r*_exec.fun_. = −0.69, *p* = 0.001), although only the executive function sub-score correlation reached statistical significance after Bonferroni-correction for multiple correlations. The executive function scores also correlated with the standard deviation of response time in the cognitive induction condition (*r* = −0.55, *p* < 0.05). Interestingly, cognitive induction scores in the visuospatial and memory domains did not correlate significantly with CPT scores (*r* = −0.05 and 0.04, respectively).

##### SST

**FIGURE 12:**
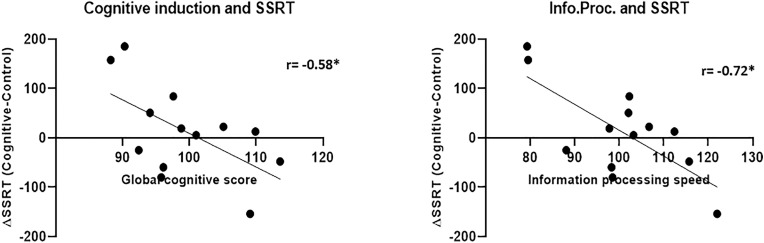
Cognitive induction measures and SSRT between-conditions difference. Better performance in the cognitive induction task, as measured by the global cognitive score **(left)**, was related to SSRT improvement (representing response inhibition) in the MPH-Cognitive condition compared with the MPH-control condition. This relationship was also found for the information processing speed subscore **(right)** (*p < 0.05, Bonferroni-corrected).

The SSRT results in both conditions did not correlate with the global cognitive score and in the case of the control condition, also with the sub-scores (Figure 12). The SSRT in the cognitive condition correlated with the information processing speed subscore (*r* = −0.53, *p* = 0.04), while the SSRT in the control condition did not correlate significantly with any of the cognitive induction scores- global or subscales. More importantly, the difference in the SSRT between the cognitive condition and the control condition, although by itself not significant as mentioned, did correlate with the global cognitive score (*r* = −0.58, *p* = 0.039) and also with the information processing subscore (*r* = −0.72, *p* = 0.006), indicating that increased cognitive induction was related to response inhibition improvement in the MPH-CogniCognitive condition compared with the MPH-control condition. It should be noted that the attention (*r* = −0.42, *p* = 0.15) and executive function (*r* = −0.45, *p* = 0.12) subscores showed a negative correlation trend as well, although not statistically significant.

### Discussion

Study 2 aimed to evaluate the clinical plausibility of the functional-pharmacological coupling strategy demonstrated in study 1 in adult-ADHD. ADHD-diagnosed participants were administered with a therapeutic dose of MPH, coupled either with a cognitive induction task or a control task. The results complement the results exhibited in study 1, by pointing to the benefit of coupling MPH treatment with cognitive stimulation in terms of sustained attention, as reflected mainly in the CPT scores. Participants were faster in reaction time, performed less omission errors, and had less variation in their response time in the MPH-CogniCognitive induction condition, compared with the control condition. There was no evidence of better response inhibition, as reflected in the SST scores. In accordance with the results found in study 1, better performance in the cognitive induction task, both globally and in specific cognitive domains, was related to better performance in the ADHD-related tasks – both the CPT and the SSRT. Interestingly, this relationship between cognitive induction and ADHD-related performance was selective for both tasks; it was evident only for domains such as executive function and information processing, and not for less ADHD-relevant domains such as memory and visuospatial performance. These findings point both to the domain-specificity of the functional-pharmacological coupling effect and to its dependence on cognitive engagement in this case.

Besides providing a clinical support to the concept of functional-pharmacological coupling, study 2 further examines its impact on specific ADHD-related domains. The first domain is *sustained attention*, which plays an important role in daily life; the ability to maintain attention while driving, during social interactions, etc., is a major component of normal cognitive functioning (Sarter et al., 2001). Deficits in sustained attention might have manifestations in the academic, professional, and social domains, decreasing quality of life and well-being. The CPT is a common quantitative measure for sustained attention (Shalev et al., 2011). A within-task distinction is made between omission errors, which have been considered a measure of sustained attention, and commission errors, which are reported as a measure of impulsivity (Epstein et al., 2010). In the current study, there was a significant difference between conditions in the omission errors measure, while no significant difference in commission errors was found. The standard deviation in response time is also a measure of consistency, reflecting sustained attention. In this measure as well, there was a significant difference between conditions, such that coupling MPH with a cognitive state resulted in reduced response time variability. The overall results provide clear evidence that coupling MPH with cognitive induction has a beneficial effect in terms of sustained attention performance.

The second domain examined in study 2 was *response inhibition*, which was measured by the SST. A smaller sample size was available compared with the CPT was available for this measure. Coupling MPH with cognitive induction did not yield better response inhibition results, contrary to our hypothesis. Considering that MPH is known to improve response inhibition in adult ADHD (Vaidya et al., 1998; Aron et al., 2003), and taking into account our sample of young adults, mostly university students, a ceiling effect might have contributed to the fact that no significant between-condition differences were found. The SSRT score between-conditions difference, however, did correlate with the extent to which the participant was cognitively inducted. The correlations found between induction measures and ADHD-related performance, in both tasks (CPT and SST), suggest that cognitive-induction parameters might affect the efficacy of functional-pharmacological coupling, represented by performance in ADHD-related measures.

This study had several limitations. First, there was no placebo control. This raises the possibility that the between-condition effects found resulted from the fact that in the cognitive induction condition, the induction served as “cognitive training.” Thus, the effects are “training-related,” rather than stemming from the unique coupling of MPH with cognitive induction. The fact that correlations were found between induction scores and SSRT improvement (considering both conditions) undermines this possibility to a certain extent, although further studies with placebo control will provide a better insight in this regard. Hence, our results should be interpreted with caution. Second, both Study 1 and Study 2 employed a rather broad induction battery, which may not be focused enough for triggering specific cognitive domains. Further studies may benefit from matching the cognitive induction to the test measure. As the activation of specific neural circuits may enhance drug delivery, a more refined functional activation pattern might induce better delivery and efficacy. Finally, considering the important use of ADHD-related measures in an ADHD sample, this clinical study did not provide mechanistic insights regarding the functional-pharmacological coupling effect of MPH. Further research should examine whether the coupling effect, found significant in this study, relies on specific neural mechanisms known to be crucial in response inhibition and sustained attention, such as frontostriatal circuitry, in general, and the right inferior frontal cortex (rIFG), specifically (Aron et al., 2004, 2014).

## General Discussion

The goal of this work was to present and examine a novel approach for improving drug efficacy in the human brain, by coupling a specific functional state with drug administration, i.e., functional-pharmacological coupling. The leading assumption was that specificity and efficacy of pharmacological agents are modulated by the concurrent functional state of the targeted brain system, which is closely related to the person’s mental state. This approach was formulated based on several foundations. Pharmacokinetic and pharmacodynamic factors, such as CBF, were shown to be state-dependent, subjected to endogenous and exogenous modulations, which alter target circuit responsiveness. In addition, a large body of research provided elaborate evidence regarding the interactions between empirically induced mental states and drug effects (Ritz et al., 1987; Overton and Clark, 1997; Volkow et al., 2001; Swanson et al., 2002). It followed that intentionally inducing a specific mental state might alter the brain’s responsiveness to an administered drug. This approach is clinically driven, with an overreaching aim to improve therapeutic neuropsychopharmacology.

A review of the current literature shows a relative paucity of research regarding state-drug interactions in clinical medicine, and the knowledge accumulated from animal studies regarding drugs of abuse has not yet been translated for the betterment of drug therapeutics. As opposed to routine instructions accompanying drug administration regarding optimal gastro-intestinal absorption (e.g., “take this medication with food”), better absorption in terms of the target system, i.e., the brain, is overlooked. Considering the dynamic nature of brain activity and connectivity, as revealed in the last few decades by neuroimaging methods, it is a critical goal to link pharmacological agents with certain functional states for better drug targeting and less adverse effects.

Our drug-of-choice for employing the functional-pharmacological coupling was MPH in the framework of the dopaminergic system. The approach was demonstrated and examined via two studies. Study 1 included an experiment with healthy participants, examining whether coupling MPH with cognitive induction will result in better executive function performance and in an altered brain activation pattern. This was performed via a complex within-subject, placebo-controlled neurobehavioral design. Study 2 included a clinical examination of the functional-pharmacological coupling of MPH with cognitive induction in ADHD-diagnosed participants, evaluating the effect on ADHD-related measures of response inhibition and sustained attention.

The objective of study 1 was to demonstrate the neurobehavioral effects of functional-pharmacological coupling. It was hypothesized that coupling MPH with cognitive state induction of the mesocortical pathway will result in a behavioral, executive function effect and a corresponding prefrontal activation effect. Coupling MPH with cognitive state induction yielded a specific brain-behavior correlation: rDLPFC recruitment was linked to improvement in the N-back task only when coupling MPH with cognitive induction, but not when coupling MPH with a mesolimbic-oriented induction or when coupling placebo with either inductions. Moreover, the more engaged the participants were in the cognitive induction, the better they performed in the task’s hardest condition. The correlations were unified to a mediation model by which cognitive induction engagement mediated the relationship between rDLPFC recruitment and enhanced N-back performance.

The objective of study 2 was to evaluate the clinical effect of MPH functional-pharmacological coupling in ADHD. It was hypothesized that MPH-Cognitive coupling will better target the drug to the mesocortical pathway, resulting in improved performance in ADHD-related measures – sustained attention and response inhibition. Study 2 provided evidence regarding the functional-pharmacological coupling translational potential. ADHD-diagnosed adults performed better in the sustained-attention task when the MPH administration was coupled with cognitive induction. In this case, there was a between-conditions effect, but not a placebo-controlled effect, such that this finding should be interpreted with caution. Similarly to Study 1, Study 2 also provides evidence that better induction was correlated with better subsequent cognitive performance (in this case, both in response inhibition and sustained attention), serving to ensure that at least two of the three coupling phases (induction and effect measurement) were correlated as hypothesized.

Functional-pharmacological coupling represents a broad concept and may relate to a multitude of pharmacological agents, induction procedures and pathophysiological entities. A major foundation of this concept is that increased blood flow to a certain region might increase drug delivery and specificity. In the current research, we did not employ methods for direct examination of this presumed effect, such as PET (Spencer et al., 2006). Future research might benefit from investigating the functional-pharmacological effect using a multi-modal approach, for example by combining PET with fMRI. Thus, both neural activity/connectivity and receptor/transporter binding will be measured simultaneously, demonstrating both functional and pharmacological effect validation.

Our correlation analyses suggest that the efficiency of functional-pharmacological coupling may depend on individual characteristics, such as engagement and performance on the induction task. Along with these psychological parameters, it is highly probable that physiological factors mediate the functional-pharmacological coupling effect as well. We hypothesize that among these factors, physiological processes related to *age* would be most influential. This hypotheses relies on accumulating evidence, suggesting that the neurovascular coupling mechanism is impaired among aged individuals (e.g., Fabiani et al., 2014; Balbi et al., 2015; Lipecz et al., 2019). Thus, for example, changes in neurovascular coupling, as probed by measuring the maximal impact of flickering the diameter of retinal arterioles, were reduced in 51.78% in aged relative to young humans (Lipecz et al., 2019). Moreover, this impairment has been associated with cognitive deficits (e.g., Tucsek et al., 2014; Tarantini et al., 2017, 2018). Interestingly, recent studies suggest that these deficits can be ameliorated by neurovascular coupling enhancers. For example, a 2-week treatment of aged mice with an inhibitor of an enzyme that cleaves NAD^+^ (the hyperactivity of this enzyme, PARP-1, in old cells in suspected to decrease NAD^+^ levels; Csiszar et al., 2019) resulted in the restoration of neurovascular coupling indices to the levels observed in young mice, as well as in an improvement in the performance of cognitive (but not non-cognitive) tasks (Tarantini et al., 2019).

Since the functional-pharmacological coupling effect assumingly relies on neurovascular coupling, its magnitude is expected to decrease with age. To address this limitation, functional-pharmacological protocols should be designed with sensitivity to age. Their difficulty levels or temporal intensity can be enhanced to compensate for age-related neurovascular coupling impairments. We also expect that the combination of such protocols with the administration of neurovascular coupling enhancers (in animals or when approved for clinical use in humans) will result in an incremental cognitive improvement beyond the gross impact of such enhancers.

This research focused on the dopaminergic system and on MPH as a pharmacological agent. A U-shaped relationship between DA levels (endogenic or pharmacologically induced) and cognitive performance (Cools and Robbins, 2004) has been suggested, showing that both low and high levels of DA may impair performance (Floresco and Magyar, 2006). This aspect should be addressed in future studies, for example by conducting a longitudinal study in which baseline cognitive performance would be more easily established per participant.

Brain activation results (study 1) should be interpreted with caution, as participants were healthy adults; increasing MPH effects via better delivery might have different results in ADHD-diagnosed individuals with abnormal basal DA levels. Furthermore, the “ADHD-brain” was shown to have distinct characteristics in terms of activation and connectivity, with psychostimulants designed to normalize dysfunctions (Konrad and Eickhoff, 2010; Castellanos and Proal, 2012) compared with healthy controls, further qualifying our results. Future research might expand the findings to include ADHD-populations (children and adults).

The selected state induction procedures were another significant methodological issue. In study 1, cognitive state induction was achieved by a rather broad cognitive battery challenging various cognitive domains (Schweiger et al., 2003; Doniger, 2013). It is possible that a more focused cognitive induction procedure would have resulted in better activation, and subsequently a better coupling effect; this applies to the control induction procedure as well. Real-time validation of induction efficacy, in terms of activating the proper neural circuits (i.e., mesocortical and mesolimbic), would be a significant advance in the functional-pharmacological coupling examination.

The sample size in this research was relatively small; this is especially significant for the data reported in study 1, which suffers from relatively low statistical power. Although the use of a within-subject, placebo-controlled design yielded a considerable amount of data and decreased inter-individual variability, the examination of functional-pharmacological coupling requires multiple control conditions and would benefit from an increased number of participants in future studies, which might provide a needed replication of the patterns exhibited here.

Finally, a generalization of the functional-pharmacological coupling concept must clearly rely on further research with other pharmacological systems, linked with other pathophysiological entities. This could be addressed by multiple hypothesis-driven functional-pharmacological coupling procedures with relevant readout measures.

### Significance and Conclusions

The efficacy of psychopharmacological treatment is often considerably restricted by the fact that drugs reach both pathologically relevant as well as irrelevant brain areas in a non-selective manner, thus causing desired but also unwanted effects. Medicine, and neuropsychiatry specifically, is currently in the midst of a vast effort to individualize the treatment of both acute and chronic diseases, for example by harnessing genetic data to try and anticipate drug response and adverse effects. Pharmacological investigation is usually designed to control for intervening context and environmental factors. Thus, drug efficacy is tested by isolating the pharmacological active ingredient’s effect, while controlling for all other contaminating artifacts. Notwithstanding, however, critical this process may be for solid empirical proofs, it embodies a cardinal blind spot: contextual factors may not only hinder pharmacological treatments, but also strengthen them, and could potentially be manipulated in order to improve clinical outcomes (Price et al., 2008).

The growing body of knowledge regarding the functional patterns of neural activity, and the realization that functional patterns are dynamic by nature, ever-changing and reacting both to internal and external stimuli, makes it more complex to characterize the exact setting to which the drug enters as an agent in the brain. Nevertheless, the characterization of brain networks as coupled functional-pharmacological entities may significantly improve the locality of drug delivery, and thus reduce their unwanted side effects. We believe that this aspect of clinical neuropsychopharmacology has been somewhat overlooked.

A bedside method of enhancing the specificity of drug delivery would have important clinical implications. First and foremost, it may allow a reduction in the dosage required for satisfactory therapeutic effects in the target area (i.e., greater efficacy), resulting in reduced abuse and an improved side effect profile. In addition, and no less important, it may result in better adherence and reduced economic burden.

The functional-pharmacological coupling concept offers a promising new direction in the daily practice of neuropharmacology, toward becoming more active and individualized. Although limited and partial, the evidence presented in this work points to the potential of coupled functional-pharmacological drug targeting, which can be further implemented to improve drug delivery in other pathological conditions.

## Data Availability Statement

The raw data supporting the conclusions of this article will be made available by the authors, without undue reservation.

## Ethics Statement

The studies involving human participants were reviewed and approved by the Tel Aviv Sourasky Medical Center’s Ethics Committee. The patients/participants provided their written informed consent to participate in this study.

## Author Contributions

GR and TH conceived the functional pharmacology concept. HS and NL supervised the project. RS-E and TH designed the experiment. RS-E and GR conducted the experiment and performed the analyses. All authors participated in interpretation of results and writing of the manuscript.

## Conflict of Interest

The authors declare that this study received funding from the Teva Pharmaceutical Industries Ltd., in the form of a scholarship to RS-E. The funder was not involved in the study design, collection, analysis, interpretation of data, the writing of this article or the decision to submit it for publication.
